# Cerebral venous thrombosis: Current management, recent advances and future directions

**DOI:** 10.1177/17474930251393311

**Published:** 2025-11-16

**Authors:** Jelle Vellema, Anita van de Munckhof, Jonathan M Coutinho

**Affiliations:** 1 Department of Neurology, Amsterdam UMC, Location University of Amsterdam, Amsterdam, The Netherlands

**Keywords:** Cerebral venous thrombosis, cerebral venous sinus thrombosis, direct oral anticoagulants (DOACs), endovascular treatment, decompressive surgery

## Abstract

**Background and aim:**

Cerebral venous thrombosis (CVT) is an uncommon but increasingly recognized cause of stroke.

Despite its lower incidence than arterial stroke, CVT can cause substantial functional disability and mortality and mainly affects younger adults. This review summarizes current treatment strategies, recent advances, and potential future directions.

**Recent advances:**

Anticoagulation remains the cornerstone of CVT treatment. While vitamin K antagonists (VKAs) have long been the standard, direct oral anticoagulants (DOACs) have recently been demonstrated to be equally safe and effective, and are increasingly used in routine practice. Endovascular therapy is reserved for selected severe cases unresponsive to anticoagulation, although data from randomized trials remain limited. The recently completed DECOMPRESS2 study has provided high-quality data on the outcomes of patients with severe CVT that underwent decompressive surgery. Novel scoring systems, such as DIAS3 and SI(2)NCAL(2)C, have helped facilitate individualized prediction of seizures and long-term outcomes.

**Future directions:**

The diagnostic work-up of CVT could be further improved if clinical decision rules, in combination with biomarkers, are developed and validated. Similarly, Artificial Intelligence algorithms that are able to detect signs of CVT on imaging, even when CVT is not suspected, could help to speed up diagnosis of CVT, allowing faster treatment. Novel anticoagulant and fibrinolytic treatments hold promise to rapidly and safely achieve recanalization of the venous system. Finally, multicenter studies should address novel ways to measure outcome after CVT, beyond the modified Rankin Scale. As with all CVT research, international collaboration through academic consortia will be the key to produce evidence-based answers to the burning clinical questions, with the ultimate goal to reduce the global burden of this condition.

## Graphical abstract

**Graphical abstract:**
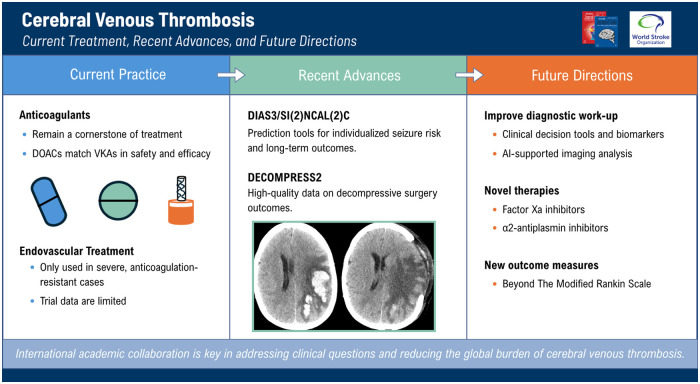

## Introduction

**Figure 1.**
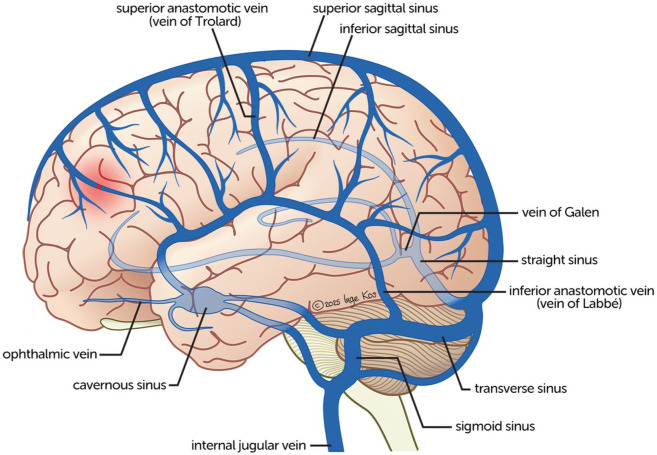
Anatomy of the cerebral venous sinuses and cortical veins.

Cerebral venous thrombosis (CVT) is a rare cause of stroke, characterized by the formation of a thrombus in the dural venous sinuses and cortical veins (Figure 1).^1^ Epidemiological studies indicate that the incidence of CVT has risen from earlier estimates of approximately 0.3 to 0.5 cases per 100.000 individuals to more recent numbers of 1.2–1.3 cases per 100.000.^2–4^ Traditionally, CVT is considered a disease of younger adults, especially women, often in the context of pregnancy or oral contraceptive use.^5,6^ But while incidence has increased across all age groups in recent decades, the most pronounced relative rise has occurred among individuals over 50 years of age, irrespective of sex.^7^ Explanations for this increase among older patients may be improved radiological detection, increased rates of obesity, overall higher survival of prothrombotic diseases such as cancer, and COVID-19-related CVT. Despite these trends, early mortality has remained relatively stable around 3%, whereas long-term sequelae—especially in the cognitive domain—are less well characterized.^8^

As with other locations of thrombosis, the pathophysiology of CVT can be understood within the framework of Virchow’s triad, encompassing endothelial dysfunction, alterations in (cerebral) venous blood flow, and a hypercoagulable state.^9^ Known risk factors for CVT include pregnancy, oral contraceptive use, genetic or acquired thrombophilia, malignancy, and autoimmune conditions. Local precipitating factors, such as central nervous system infections, traumatic brain injury (especially with accompanying skull fractures), and jugular venous catheters, can also cause CVT. (Ferro, Canhão, Stam, Bousser and Barinagarrementeria, 2004) During the COVID-19 pandemic, SARS-CoV-2 infection emerged as risk factor for CVT, as did—in rare cases—vaccination with adenovirus-based COVID-19 vaccines.^10,11^

Clinical features of CVT can generally be classified into four major clinical syndromes.^12^ First, isolated intracranial hypertension, caused by increased venous pressure and impaired cerebrospinal fluid absorption, typically manifests with severe headache, visual disturbances, nausea, vomiting, and papilledema. The second is a focal syndrome, reflecting parenchymal injury, and presenting with focal neurological deficits such as hemiparesis or aphasia, often accompanied by epileptic seizures. The third is encephalopathy, characterized by altered mental status or coma, which can be due to thrombosis of the deep venous system or large venous hemorrhagic infarcts. Finally, cavernous sinus thrombosis presents with periorbital pain with palsy of cranial nerves III, IV, V, and VI.^12,13^ These syndromes are not mutually exclusive, but may also present concurrently.^14^

Neuroimaging is required to confirm or exclude CVT. Non-contrast-enhanced computed tomography (NCCT) is often the initial imaging modality and may reveal hyperdensity of the affected venous structures or an associated juxtacortical hemorrhage.^15^ When relying on CT imaging, CT venography is required for a definitive diagnosis, by demonstrating intraluminal filling defects at the site of thrombosis.^16^ Magnetic resonance imaging (MRI) combined with MR venography is considered the gold standard, offering superior sensitivity for cortical vein thrombosis and more detailed assessment of parenchymal lesions.^17^

Due to its low incidence, CVT has always been challenging to study. Nonetheless, significant progress has been achieved, largely through extensive international collaboration, leading to better epidemiological insights, therapeutic advances and improved management of complications. This collaborative framework also provides a robust foundation for future research. In this review, we summarize recent scientific insights and provide suggestions for future studies that can help to improve the prognosis of patients with CVT, thereby reducing the global burden of this condition. In order to broadly capture clinically relevant advances, we performed a PubMed search covering the past 5 years, screening titles and abstracts of all multicenter studies reporting original data.

## Recent advances

### Epidemiology

Risk factors for CVT are very diverse and can be either transient or persistent. Some risk factors for CVT are related to thrombosis in general, such as thrombophilia, while others are more specifically related to CVT, like head and neck infections. Female-specific risk factors play an important role in the development of CVT, and a recent systematic review found that postpartum CVT is estimated to occur in 7 per 100,000 deliveries.^18^ The risk of pregnancy-related CVT is higher for women with a history of CVT (i.e. 10.2 per 1000 deliveries), emphasizing the need of treatment optimalization in this group.

In 2021, CVT was found to be a rare complication of adenovirus-based SARS-CoV-2 vaccines. As a result of anti-PF4 antibodies that activate thrombocytes, these patients developed extensive thrombosis, often located in the cerebral venous system, in combination with thrombocytopenia. The condition was named Vaccine-induced Immune Thrombotic Thrombocytopenia (VITT). CVT due to VITT was estimated to occur after 4 to 16 per million first-dose vaccines with the highest risk after the ChAdOx1 nCov-19 (AstraZeneca) vaccine.^19,20^ This severe complication was most commonly seen in younger patients and had a mortality rate up to 50% in the acute phase,^19,21^ which led to restrictions on the use of these vaccines in many countries. Specific treatment strategies were proposed, but use of immunomodulation was found to be most effective.^22^ Over time, the mortality rate lowered and the long-term outcomes in patients who had survived the acute phase appeared to be favorable.^23^ The exact pathophysiology of VITT and why there was a predisposition for thrombosis of the cerebral veins specifically has unfortunately never been fully clarified.

### Diagnosis

CVT can be difficult to diagnose due to its rarity and heterogeneity in presenting symptoms. A recent study showed that the majority of patients sought medical care in the week prior to the CVT diagnosis,^24^ suggesting that signs of CVT may initially be missed by healthcare professionals. Early diagnosis of CVT and timely initiation of treatment can prevent complications such as venous infarction or intracerebral hemorrhage. In a retrospective cohort of CVT patients, isolated headache and older age were characteristics associated with a delayed diagnosis of CVT.^25^ In this study however, delayed diagnosis was not associated with a worse clinical outcome, but these results should be interpreted with caution, given the inherent bias of such analyses. Several laboratory tests, like D-dimer, and new radiology techniques have been proposed to improve diagnosis of CVT, but their value remains to be established in large, multicenter studies.

### Treatment

Anticoagulation remains the cornerstone in treating CVT. After an initial course of heparin, patients are usually transitioned to oral anticoagulation. For many years, vitamin K antagonists (VKAs) were the oral anticoagulant of choice, and until recently, it was unclear whether direct oral anticoagulants (DOACs) had a similar efficacy and safety. In the last 5 years, the evidence for use of DOACs to treat CVT has substantially grown. Following the publication of RESPECT-CVT in 2019,^26^ the randomized SECRET, CHOICE-CVT, and RWCVT trials were published on efficacy and safety of DOACs versus VKAs.^27–29^ In addition to these small trials, the large prospective DOAC-CVT study and retrospective ACTION-CVT study were also published.^30,31^ Based on all available data, DOACs appear to have similar effectiveness and safety as VKAs to treat CVT. Of note, since only limited data are available on direct initiation of DOACs, initial treatment with heparin for several days remains the standard.^32^

A major topic of interest regarding treatment of CVT is the use of endovascular therapy (EVT). The TO-ACT trial, published in 2020, did not show benefit of EVT in patients with severe CVT.^33^ Nevertheless, a recent survey about practice patterns and perspectives on the use of EVT showed that three out of four respondents supported use of EVT for CVT in certain clinical situations, such as worsening level of consciousness.^34^ In recent years, a number of new studies on EVT have been published.^35–40^ The results of these studies regarding improvement of functional outcomes after EVT are conflicting, emphasizing the need for further controlled studies on patient selection and techniques for EVT to treat CVT.

### Complications

International guidelines recommend to use decompressive surgery in patients with severe CVT and impending herniation due to parenchymal lesions. In 2024, the DECOMPRESS2 study was published that reported on 118 patients with severe CVT that all underwent decompressive surgery. This prospective, single-arm, observational, international cohort study provides comprehensive data on the outcome of this subset of patients with severe CVT. More than half of the patients were comatose at baseline and a quarter had fixed and dilated pupils. One year after treatment, two-thirds of patients were alive and one-third of patients were functionally independent (modified Rankin Scale [mRS] score 0–2). In a subanalysis of DECOMPRESS2 on timing of anticoagulation after decompressive surgery, 2 out of 5 patients started anticoagulation within 24 h after surgery.^41^ The timing of anticoagulation initiation after decompressive surgery did not seem to be associated with outcomes.

CVT is complicated by seizures in 20–35% of patients in the acute phase.^42,43^ Acute seizures are associated with higher disability and mortality.^43^ Factors associated with acute seizures are intracerebral hemorrhage, parenchymal lesions, thrombus located in the superficial system, and focal neurological deficits.^42,44,45^ Late seizures are defined as seizures occurring more than 7 days after CVT diagnosis and affect 1 in 10 patients with CVT.^46^ Several predictors for late seizures have been identified, including acute seizures, intracranial hemorrhage at presentation, decompressive hemicraniectomy, and age.^44,46,47^ Based on these predictors, the DIAS3 score was developed to predict the risk of post-CVT epilepsy for individual patients, aiding in decision-making regarding treatment with antiepileptic drugs and patient counseling.^47^

### Prognosis and long-term outcomes

In recent years, several scores have been developed for outcome prediction in patients with CVT. A tool that has been developed to predict mortality and poor outcome is the SI(2)NCAL(2)C score based on nine predictors.^48^ This score had an area under the curve (AUC) of 0.80 for poor outcome, 0.84 for 30-day mortality, and 0.84 for 1-year mortality. The robustness of the SI(2)NCAL(2)C score was shown in an external validation study, yielding similar results.^49^ The IN-REvASC Score was derived from the ACTION-CVT cohort and includes seven items associated with poor outcome, defined as an mRS score of 3 to 6.^50^ The score had an AUC of 0.84 for prediction of poor outcome at 90 days.

The risk of recurrent thrombosis after CVT is estimated at 1–3% per year.^51^ Several factors have been associated with a higher risk of recurrent thrombosis, such as a history of venous thromboembolism (VTE).^51,52^ In line with studies on deep vein thrombosis, CVT etiology could also be a predictor for the risk of thrombotic recurrences. A recent study found that patients with persisting risk factors for CVT had a more than two-fold risk of recurrent thrombosis compared to patients with transient provoking factors.^53^ Identifying subgroups of CVT patients with high risks of thrombotic recurrences might be relevant to improve individualized treatment strategies, including low-dose DOACs, which are now routinely used to reduce the risk of recurrent thrombosis after VTE.^54^

An important insight regarding outcomes after CVT was the recent report from three nationwide studies that patients with CVT have an increased risk of a new cancer diagnosis compared to controls, especially in the first year after CVT.^55–57^ In these studies, the absolute risk of a new cancer diagnosis after a first episode of CVT increased with age, but the relative risk was highest in younger patients.^55–57^ While it remains uncertain whether systematic screening for cancer after CVT is beneficial, these results do warrant vigilance for signs or symptoms of cancer during the follow-up of patients with CVT.

## Future directions

### Diagnosis

**Figure 2.**
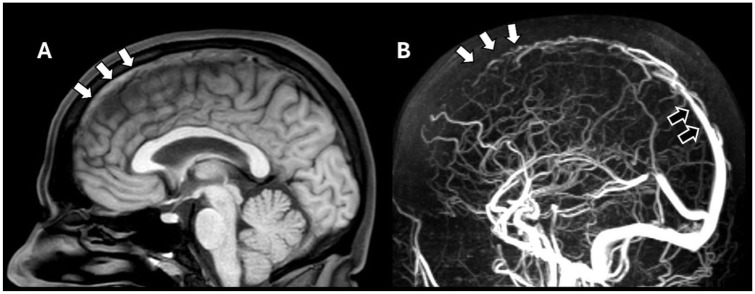
(a) Mid-sagittal T1 weighted MRI showing hyperintensity in the anterior superior sagittal sinus, indicating fresh thrombus within the venous sinus (white arrows). (b) Sagittal MR-venogram of the same patient. There is absence of flow within the anterior superior sagittal sinus, confirming there is a thrombus in this vein (white arrows). For reference, normal opacification is seen in the posterior segment of the superior sagittal sinus (black arrow).

Under-recognition of CVT at initial presentation is an important clinical challenge, which can lead to a delay of several days between symptom onset and diagnosis.^6,30^ The variability in clinical presentation makes it difficult for physicians to timely recognize CVT, especially because suspicion of the disease can be raised in highly variable health care settings and a contrast-enhanced CT or an MRI is needed to confirm or rule out the disease (Figure 2). Furthermore, CVT patients are commonly examined first by general practitioners and ER physicians who may lack experience with CVT, as it is an uncommon disease. In certain patients, clinical suspicion of CVT is relatively high, particularly when headache is accompanied by seizures or focal neurological deficits. Diagnostic uncertainty is greatest in patients presenting with isolated headache, as headache is one of the most common complaints in the emergency department and the prevalence of CVT in this population is obviously very low.^25,58^

Earlier diagnosis of CVT allows prompt initiation of anticoagulation, which is important to achieve venous recanalization. Recent studies have demonstrated that early recanalization reduces the chance of brain damage and improves clinical outcome.^59^ In addition, rapidly ruling out CVT in low-risk patients could make the work-flow in the hospital more efficient and prevent unnecessary imaging. There are two particular innovations that would greatly help to speed up diagnosis of CVT: (1) An easy-to-use decision algorithm that helps differentiate CVT from mimics; (2) Artificial Intelligence (AI)-supported algorithms that can automatically help detect CVT on routine brain imaging, even when CVT is not suspected by clinicians.

In patients with suspected VTE in more common sites such as deep venous thrombosis and pulmonary embolism, the Wells criteria, based on clinical features that can be easily ascertained at the patients’ bedside, in combination with D-dimer values, have greatly facilitated accurate diagnosis because it allows risk stratification and abolishes the necessity for imaging in many patients.^60^ A similar validated diagnostic tool, which is currently not available, would greatly assist the diagnostic work-up of patients with suspected CVT as it would allow rapid triage and select only those at high risk of CVT for ancillary contrast-enhanced CT or MRI. In 2020, a collaboration of researchers of the international CVT consortium developed a clinical score (Table 1) which, in combination with D-dimer values, holds promise to predict CVT.^61^

**Table 1.** CVT clinical score.

Subitems	points
Epileptic seizure(s)	4
Known thrombophilia	4
Oral contraception	2
Duration of symptoms > 6 days	2
Worst headache ever	1
Focal neurological deficits	1

Source: From Heldner et al.^61^ Neurology (2020).

0–2 points: low CVT probability. 3–5 points: moderate CVT probability. 6–14 points: high CVT probability.

The AUC in the inception cohort of 359 patients with suspected CVT was 0.94 (95% CI 0.91–0.96).^61^ This study, however, had a limited sample size, used data of only two hospitals, and lacked an independent validation cohort. Prior to implementation in routine practice, these results need to be confirmed in an independent, large, prospective, multicenter study. In the longer term, novel blood-based biomarkers of thrombosis activation and neuroglial injury, such as P-selectin, glial fibrillary acidic protein (GFAP), brain-derived Tau, and neurofilament light, may further optimize the diagnostic work-up of CVT.

**Figure 3.**
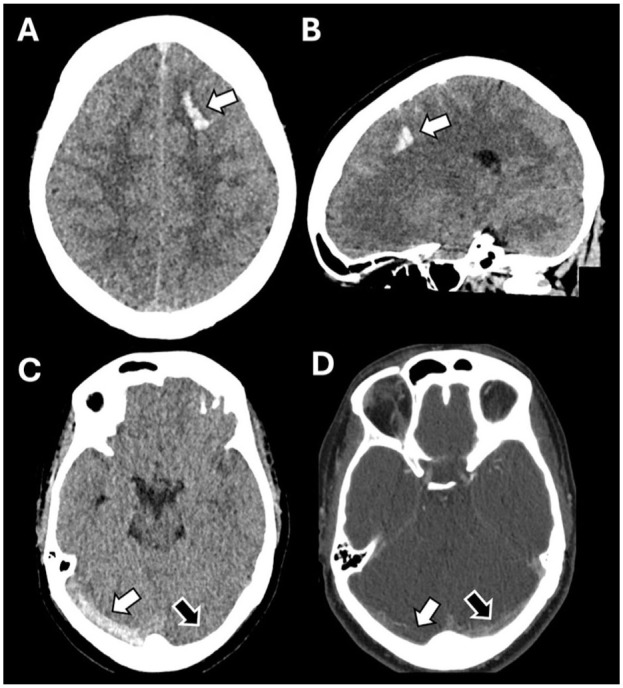
Non-contrast CT images of CVT patients with markers that could help to identify CVT using artificial intelligence. (a/b) A solitary juxtacortical hemorrhage is situated in the frontal lobe of the left hemisphere, confined to the white matter just below the cortex. (c) Patient with a right transverse sinus thrombosis and corresponding dense vessel sign (white arrow). The left transverse sinus demonstrates normal density (black arrow). (d) Contrast-enhanced CT of patient C, with the white arrow indicating the thrombosed sinus and the black arrow demonstrating preserved vessel patency.

In routine clinical practice, patients with severe headache are often first evaluated with NCCT, which, to date, is insufficient to reliably diagnose or exclude CVT. Nevertheless, NCCT may demonstrate subtle imaging findings suggestive of CVT, such as hyperdense sinuses or cortical veins (cord sign), subarachnoid hemorrhages, and small juxtacortical hemorrhages (cashew nut sign; Figure 3).^15,16^ A recent study among 88 patients with suspected CVT found a sensitivity of 77% and specificity of 93% to diagnose CVT based on sinus attenuation on NCCT.^62^ Another study among 201 patients found a sensitivity of 91% when attenuation was combined with hematocrit values.^63^ At initial presentation, these signs are often missed due to their subtle nature and limited recognition among clinicians and radiologists. An AI–based radiological support tool could greatly enhance routine care for patients with suspected CVT. Upon acquisition of NCCT, such an algorithm could rapidly alert clinicians to the possibility of CVT, even before it is clinically suspected. Such a warning could prompt clinicians to conduct contrast-enhanced imaging to confirm the diagnosis, thereby reducing the risk of missed cases and reduce time to treatment.

### Treatment—medication

There are no data from CVT patients that suggest that one particular DOAC is preferable, although dabigatran and apixaban appear to be used most often.^30^ Recent meta-analyses of VTE treatment indicate that while all DOACs demonstrate broadly comparable efficacy in preventing recurrent VTE, their safety profiles appear to differ.^64^ Notably, apixaban is associated with the lowest risk of major and clinically relevant non-major bleeding among the available agents.^64,65^ These mostly indirect comparisons were recently confirmed by the results of the COBRA randomized controlled trial, which directly compared apixaban to rivaroxaban in patients with VTE.^66^ Among 2760 patients treated, apixaban was associated with a lower incidence of combined major and clinically relevant non-major bleeding compared to rivaroxaban (3.0% vs 6.7%, respectively) during 3 months of treatment. Direct extrapolation of these results to CVT should of course be done with caution. Ideally a randomized trial would be performed in patients with CVT, but this is likely infeasible due to the rarity of the condition. However, pooled data from recent retrospective and prospective studies comparing warfarin and DOACs could provide valuable insights into the relative efficacy and safety of the different DOACs for CVT.^27,28,30,31^

The optimal duration of anticoagulant treatment in CVT remains an area of uncertainty. Current guidelines recommend treatment for 3–12 months. In patients with recurrent thrombosis or underlying prothrombotic diseases with a high VTE recurrence risk, indefinite anticoagulation should be considered.^67^ The EXCOA-CVT cluster randomized trial directly compared a short anticoagulation strategy (⩽6 months) with an extended regimen (12 months). (Miranda, Aaron and Arauz, 2018) The 12-month follow-up data, presented at the European Stroke Organisation Conference 2025, demonstrated no advantage of extended treatment with respect to recurrent VTE or major bleeding, suggesting that routine prolonged anticoagulation may not be warranted. However, the 2-year follow-up data, which is the primary endpoint, needs to be awaited before definitive conclusions can be drawn.

In patients with deep vein thrombosis, decision on the duration of anticoagulant treatment nowadays routinely incorporates the underlying risk profile.^69^ For patients with transient risk factors such as surgery, trauma, immobilization, or pregnancy, anticoagulation is typically discontinued after 3 months, as recurrence risk is low once the provoking factor resolves.^70^ In contrast, patients with unprovoked VTE or with persistent risk factors, such as active malignancy, and severe thrombophilia, generally require extended or indefinite therapy, as their substantially higher recurrence risk outweighs the bleeding risk of long-term anticoagulation.^71^ Interestingly, the recently published HI-PRO trial demonstrated that among patients with VTE provoked by a transient risk factor but accompanied by a persistent moderate risk factor, such as autoimmune disease, chronic lung disease, or obesity, extended anticoagulation with low-dose apixaban for 12 months significantly reduced the risk of recurrent VTE (1.3% vs 10.0% with placebo) while maintaining a low incidence of major bleeding.^72^ These results may increase the use of low-dose DOACs after VTE in the future.

More studies among CVT patients that differentiate between risk of recurrence based on the risk factor profile (transient, persistent, and unprovoked) are needed. If a stratification pattern similar to that observed in VTE emerges, this would provide the rationale for conducting two subsequent studies: First, if the recurrence risk is low in the transient risk factor group, it could be investigated whether 3 months of anticoagulation is sufficient, as in CVT most venous recanalization occurs within 90 days after diagnosis.^59^ If these findings are confirmed, further research could investigate whether patients with concomitant persistent moderate risk factors might similarly benefit from extended anticoagulation with low-dose DOACs, as observed in the HI-PRO study. Second, studies should assess whether patients with persistent risk factors or unprovoked CVT might benefit from extended anticoagulation, and whether specific subgroups could also achieve favorable outcomes with low-dose DOAC therapy.

**Figure 4.**
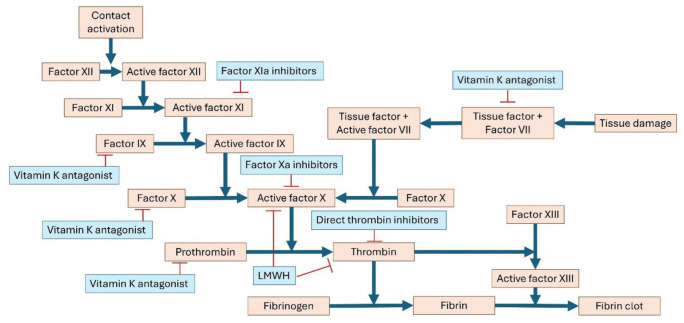
Schematic representation of the coagulation cascade. Anticoagulant drug classes and their sites of action are shown in blue. Blue lines denote activation, and red lines denote inhibition.

**Figure 5.**
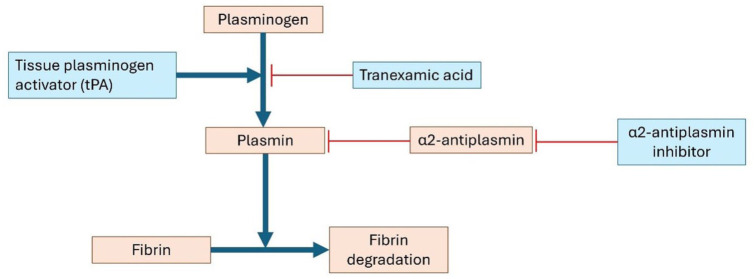
Schematic representation of fibrinolysis. In blue the different fibrinolytics and their antagonist. Blue lines indicate stimulation, whereas red lines indicate inhibition.

Despite the existing treatment options aimed at dissolution of the thrombus and recanalization of the cerebral venous system, major bleeding—especially intracranial hemorrhage—remains a risk. Two novel anticoagulant therapies that are currently undergoing clinical testing may attribute this unmet need. First, unlike DOACs, which inhibit a single coagulation protease in the common pathway to block thrombin and fibrin formation, Factor XI inhibitors only act on the intrinsic pathway (Figure 4). By selectively attenuating thrombus propagation while sparing primary hemostasis, these drugs are hypothesized to prevent pathological thrombosis with a lower incidence of bleeding.^73^ In a recent trial, abelacimab was associated with fewer bleeding complications compared to rivaroxaban in patients with atrial fibrillation, and meta-analyses suggest a trend toward reduced bleeding with Factor XI inhibition.^74,75^ Whether this safety advantage of factor XI inhibitors is also associated with antithrombotic efficacy remains to be seen, as large phase III trials in VTE are currently underway. Another novel therapeutic approach that is being investigated to safely dissolve venous thrombi without increasing bleeding risk, is through targeting fibrinolysis inhibitors such as α2-antiplasmin (Figure 5).^76^ It is known that elevated levels of α2-antiplasmin contribute to thrombus resistance to fibrinolysis and that this is a risk factor for VTE.^77^ Early experimental studies demonstrated that inhibition of α2-antiplasmin accelerates thrombus resolution by enhancing plasmin-mediated fibrin degradation. Due to its specificity, this approach neither reduced circulating fibrinogen nor enhanced bleeding in a controlled hemostatic challenge.^78^ Two ongoing Phase II studies are testing monoclonal α2-antiplasmin inhibition in the treatment of acute deep vein thrombosis and pulmonary embolism (ClinicalTrials.gov identifier NCT05408546; NCT06149520). A parallel Phase II trial in acute ischemic stroke is evaluating this strategy as an acute treatment in patients with favorable perfusion profiles who are ineligible for alteplase or endovascular therapy (NCT05948566). If the studies on α2-antiplasmin and factor XI inhibitors demonstrate favorable results in more common thrombotic conditions, these novel therapies should be evaluated in patients with CVT, where major bleeding remains one of the biggest concerns.

### Treatment—EVT

Despite the neutral results of the TO-ACT trial, EVT offers a clear theoretical advantage over medical therapy alone, as it is capable of achieving venous recanalization within hours rather than days.^33,59^ The critical unanswered questions remain whether current EVT techniques—which are not developed specifically for the cerebral venous system—are suitable to efficiently and safely recanalize the cerebral sinuses, and, if so, which patient groups benefit from this therapy.

A particularly promising approach is being investigated in the ongoing PISTE-CVT study, which seeks to translate perfusion imaging techniques established in ischemic stroke to the setting of CVT. By assessing perfusion parameters on baseline MRI, investigators aim to determine whether patients with CVT can be stratified according to their risk of progression to irreversible infarction. Those identified as being at risk of developing irrevocable parenchymal lesions may represent a subgroup in whom more aggressive therapies, like EVT, could offer meaningful clinical benefit. Another interesting direction, already successfully explored in arterial ischemic stroke, is the assessment of collaterals. A small retrospective study has suggested that the presence of favorable venous collaterals is associated with favorable clinical outcomes, whereas patients with poor collaterals are at increased risk of neurological deterioration.^79^ Therefore, it can be hypothesized that patients with poor baseline collateral circulation may derive particular benefit from early recanalization.

An important consideration for future EVT trials in CVT is the definition of favorable clinical outcome. The most widely used measure of disability in cerebrovascular diseases is the mRS score. A score on the mRS of 0–1 indicates recovery without any disability and is generally regarded as a favorable functional outcome.^80^ However, this scale is suboptimal for assessing outcomes in patients with CVT, as scores, unlike in acute ischemic stroke, are often clustered between 0 and 2, reducing its discriminatory capacity. In addition, the scale lacks sensitivity to more subtle sequelae such as cognitive deficits and persistent headache which are frequently present in CVT.^81,82^ Given the relatively young age of the CVT population and the high functional demands of this life stage, the mRS alone may be insufficient. A more comprehensive approach could include detailed neuropsychological assessment, headache tests, depression scales, and return-to-work rates.

Novel devices that allow faster and more effective thrombus removal from the cerebral venous system may further enhance the efficacy of EVT. At the time of the TO-ACT trial, conventional aspiration catheters were typically 2–3 mm in diameter, and the largest stent retrievers measured around 6 mm, whereas the superior sagittal sinus averages approximately 10 mm.^83^ Consequently, complete thrombus removal was often not achievable. Currently, novel thrombectomy devices are under investigation. A recent pilot randomized clinical trial involving 61 patients undergoing EVT with a larger, next-generation device demonstrated that, compared with older devices, it did not increase complication rates and was associated with a significantly higher rate of complete recanalization.^84^ In addition, emerging imaging biomarkers may identify patients with a higher chance of successful EVT. For example, a small prospective study proposed that the presence of an acute clot sign on black blood MRI, which suggests recent thrombus formation, may predict the likelihood of successful recanalization after EVT.^85,86^ Together, these advances may facilitate a better technical performance of EVT in patients with CVT in the future.

Overall, the success of future EVT trials in CVT will hinge on the identification of factors that not only predict poor prognosis, but are also associated with an EVT treatment effect, and the adoption of more comprehensive outcome measures. Advances in these areas, combined with growing expertise of interventionalist and the development of venous-specific EVT devices, are expected to establish a clearer framework for the clinical application of EVT in the near future. The ongoing multicenter ESCORT trial in China, which enrolls patients with intracranial pressure ⩾25 cm H_2_O as the main inclusion criterion, is anticipated to provide new insights, with results expected in 2029.

### Life after CVT

While the mortality of CVT has decreased over the years,^87^ several small-scale studies indicate that many CVT survivors suffer from chronic and debilitating symptoms, such as headache, fatigue, and concentration deficits.^88,89^ The frequency and consistency of chronic symptoms in these studies suggest the existence of a “post-CVT syndrome,” similar to the post-thrombotic syndrome seen in patients after a leg-vein thrombosis. While these studies clearly suggest that post-CVT syndrome commonly occurs, their small size limits the validity and generalizability of the data. Further data on post-CVT syndrome are needed, as patient organizations identify this as a major source of uncertainty for patients and their families. A deeper understanding of its prevalence, mechanisms, and risk factors could inform strategies to mitigate its impact.

## Conclusion

Although rare, CVT is a potentially life-threatening condition and a major cause of morbidity, particularly among young adults. Recent therapeutic advances, such as the introduction of DOACs, have facilitated safer and more convenient anticoagulation strategies. Furthermore, an enhanced understanding of secondary complications, including seizure management and the role of decompressive surgery in malignant intracranial hypertension, has contributed to improved individual patient management. Future progress is anticipated through clinical decision tools, accelerated diagnostic algorithms, novel anticoagulation and fibrinolytic therapies and the expanding application of EVT. Owing to the rarity of CVT, sustained international collaboration is critical to advance knowledge and optimize patient care. Investigators and clinicians interested in contributing to these efforts are invited to join the international CVT consortium at www.cerebralvenousthrombosis.com.
